# Establishment and Evaluation of a Stable Cattle Type II Alveolar Epithelial Cell Line

**DOI:** 10.1371/journal.pone.0076036

**Published:** 2013-09-26

**Authors:** Feng Su, Xin Liu, Guanghui Liu, Yuan Yu, Yongsheng Wang, Yaping Jin, Guangdong Hu, Song Hua, Yong Zhang

**Affiliations:** 1 College of Veterinary Medicine, Northwest A&F University, Yangling, Shaanxi, China; 2 Key Laboratory of Animal Biotechnology, Ministry of Agriculture, Northwest A&F University, Yangling, Shaanxi, China; Washington State University, United States of America

## Abstract

Macrophages and dendritic cells are recognized as key players in the defense against mycobacterial infection. Recent research has confirmed that alveolar epithelial cells (AECs) also play important roles against mycobacterium infections. Thus, establishing a stable cattle AEC line for future endogenous immune research on bacterial invasion is necessary. In the present study, we first purified and immortalized type II AECs (AEC II cells) by transfecting them with a plasmid containing the human telomerase reverse trancriptase gene. We then tested whether or not the immortalized cells retained the basic physiological properties of primary AECs by reverse-transcription polymerase chain reaction and Western blot. Finally, we tested the secretion capacity of immortalized AEC II cells upon stimulation by bacterial invasion. The cattle type II alveolar epithelial cell line (HTERT-AEC II) that we established retained lung epithelial cell characteristics: the cells were positive for surfactants A and B, and they secreted tumor necrosis factor-α and interleukin-6 in response to bacterial invasion. Thus, the cell line we established is a potential tool for research on the relationship between AECs and *Mycobacterium tuberculosis*.

## Introduction

Bovine tuberculosis (BTB) is a chronic disease caused by *Mycobacterium bovis*, a member of the *Mycobacterium tuberculosis* complex, and characterized by the formation of granulomas in tissues and organs, most significantly in the lungs, lymph nodes, and intestine [1,2]. BTB is widely distributed throughout the world and causes great economic losses in animal production, especially in cattle [2-4]. Thus, a study of BTB pathogenesis in cattle is necessary and significant. Alveolar macrophages and lung epithelial cells are the first cells that encounter BTB during primary infection. Type II alveolar epithelial cells (AEC II cells) can produce relevant innate immune system molecules [5-7]. Recent research has also shown that AECs are able to internalize and control bacterial growth and present antigens to primed T cells [6]. Creating stable cattle AEC lines is thus significant for basic BTB research.

AECs are abundant and line the pulmonary airways and alveoli. AECs are composed of two types of cells. Type I AECs (AEC I) are the epithelial components of the thin air-blood barrier and comprise approximately 95% of the alveolar surface area [8,9]. Type II AECs (AEC II) cover approximately 4% of the mammalian alveolar surface and perform a variety of important functions within the lung, including regulation of surfactant metabolism, ion transport, and alveolar repair in response to injury. AEC II cells also present antigens to CD4+ T cells by expressing major histocompatibility complex (MHC) class II molecules [10-14]. AEC II cells can release a number of antimicrobial molecules, cytokines, and chemokines, including tumor necrosis factor (TNF)-α and interleukin (IL)-6, that contribute to the migration of monocytes and macrophages to the infection site and promote activation of their antimicrobial activity when bacteria invade [15]. Purification of AEC II cells is difficult, as they comprise only 15% of all lung cells. To date, no healthy cattle cell line that exhibits the full range of known AEC II functions has yet been developed [6].

Telomeres protect chromosomes from end-to-end fusion, degradation, and recombination and are therefore crucial for genome stability, cell growth control, and carcinogenesis [16,17]. The onset of replicative senescence is in part associated with the shortening of telomeres. Normal somatic cells, such as epithelial cells, are incapable of indefinite proliferation because their life span is limited by cellular senescence. Previous studies have confirmed that shortened telomeres may be the main cause of cellular senescence. As cells proliferate, their telomeres become progressively shorter so that they cannot protect the end of linear chromosomes from nuclease degradation, interchromosomal fusion, and improper recombination. As a result, the cells become senescent. Induction of telomerase activity may be a good strategy for reducing cell senescence by preventing telomere shortening [18,19]. In this respect, overexpression of human telomerase reverse transcriptase (HTERT) in cells not only prevents telomere shortening but also initiates telomerase activation and extends the life span of cells [19-21].

The current study focuses on the isolation of cattle AEC II cells and the establishment of an immortalized cell line by transfection of a plasmid containing the HTERT gene.

## Materials and Methods

### Ethics statement

All animals were handled in strict accordance with good animal practices as defined by the relevant national and/or local animal welfare bodies. The entire experimental procedure was approved by the Animal Care and Use Committee of Northwest A&F University, China, and performed in accordance with animal welfare and ethics guidelines.

### Primary culture of AEC II cells

Holstein cattle lung was excised from a healthy 90-day-old fetus. The fetus was spontaneously aborted in response to startling at the animal experiment center of the university. The tip of the lung tissue samples was then cut into 1 mm^3^ pieces. The tissues were cultured in DMEM-F12 containing 10% fetal bovine serum (Gibco, Invitrogen, Carlsbad, CA, USA), 1% (v/v) insulin–transferrin–selenium (Sigma, St. Louis, MO, USA), and epidermal growth factor (10 ng/mL; Sigma) using the tissue explant adherence method. The fibroblasts were then scraped with a scalpel until AEC II clone formation. After AEC epithelial cells clones had formed and expanded, CD74 was used as a specificity marker for AEC II fluorescence-activated cell sorting (FACS). AEC II clones were then labeled with FITC-CD74 antibody (Cat: sc-47742; Santa Cruz Biotechnology, Santa Cruz, CA, USA) and purified by FACS as previously reported [6]. In this experiment, bovine fetal fibroblast (BFF) cells were also derived from the lung tissue.

### Immunofluorescence of AEC II markers

Cells (AEC II and BFF) cultured on eight-well slide chambers were fixed with 4% paraformaldehyde and were treated with 1% Triton X-100 in PBS for 5 min. The slides were then incubated with a 1:1000 dilution of primary antibodies against cytokeratin 18 (Cat: sc-32329; Santa Cruz Biotechnology), cytokeratin 19 (Cat: sc-374386; Santa Cruz Biotechnology), vimentin (Cat: sc-373717; Santa Cruz Biotechnology), thyroid transcription factor 1 (TTF-1) (Cat: sc-13040; Santa Cruz Biotechnology), surfactant protein A (SP-A) (Cat: sc-13977; Santa Cruz Biotechnology), and surfactant protein B (SP-B) (Cat: sc-13978; Santa Cruz Biotechnology) for 2 h at 37 °C after blocking with immunostaining blocking buffer (Beyotime, Jiangsu, China) for 30 min. Slides were then incubated with a mixture of FITC-labeled goat anti-mouse IgG (Cat: A0568; Beyotime) and Cy3-labeled goat anti-rabbit IgG (Cat: A0516; Beyotime) at 37 °C for 1.5 h. The slides were observed using a laser-scanning confocal microscope (Nikon Inc., Melville, NY, USA).

### Cell transfection and selection

The pCI-neo-HTERT plasmid was kindly provided by Y.P. Jin (College of Veterinary Medicine, Northwest A&F University). AEC II cells were plated after two passages into six-well dishes and cultured in DMEM/F12 medium without antibiotics overnight. The cells were then transfected with pCI-neo-HTERT using Lipofectamine 2000 (Invitrogen) according to the manufacturer’s instructions until the cells reached 90% confluence. The medium was changed to fresh DMEM-F12 after transfection for 4 h, and the transfected cells were selected with 500 µg/mL G418 (Sigma) in complete culture medium after 24 h. Monoclonal cells appeared after 7 d of selection. One of three clones was then expanded by further culture and tested in subsequent studies. The rest of the clones were frozen in liquid nitrogen.

### Cell purity and proliferation assays

Cell purity and periodic measurement were performed by flow cytometry. AEC II markers (FITC-CD74 antibody) (Cat: sc-47742; Santa Cruz Biotechnology) were stained by immunofluorescence, after which cell purity was measured by flow cytometry (BD). Cell proliferation assays were performed as previously reported [22]. Cells were fixed and then stained by PI (Sigma), after which cycles were measured by flow cytometry. Each test was repeated three times.

### Karyotype analysis

Chromosomes were analyzed in actively proliferating cultures of transfected cells. Karyotype analysis was performed using the method reported by He et al. [22].

### Telomerase activity assay

AEC II, A549 (a lung adenocarcinoma cell line), and immortalized AEC II monoclonal cells obtained at passage 30 were prepared prior to the detection test. Telomerase activity was detected by a TRAP-silver staining telomerase detection kit (KeyGEN, Nanjing, China) according to the manufacturer’s instructions.

### Nude mice analysis

To determine the functional tumorigenicity of HTERT-immortalized AEC II cells, six 4-week-old BALB/c nu/nu mice purchased from the Shanghai Institute of Tumor Research (Shanghai, China) were housed under specific pathogen-free conditions. A tumorigenicity assay was performed by subcutaneously injecting HTERT-AEC II cells obtained at passage 50 (10^6^ cells per mouse, inoculating 3 mice) and A549 cells (10^6^ cells per mouse, inoculating 3 mice) into the 4-week-old nude mice. As a positive control, A549 cells at the same concentration were injected into the flanks of three other nude mice. The mice were observed weekly for 2 mo before they were sacrificed.

### Real Time-PCR analysis

Total RNA was extracted from fibroblasts, primary AEC II cells, and HTERT-AEC II cells at passages 5, 10, 30, and 50 using Trizol reagent (Sigma) according to the manufacturer’s instructions. cDNA was synthesized using a PrimeScript^®^ RT reagent kit (Takara, Dalian, China). The primer sequences were designed cross-intron, and PCR amplification product sizes are described in Table 1. RT-PCR results of SPA, SPB, and SPC were used for the evaluation of HTERT-AEC II features. Real-time PCR analysis was performed using SYBR® Premix Ex Taq™ (Tli RNase H Plus) according to the manufacturer’s instructions in an ABI Fast real-time PCR instrument. Fold-changes in target gene expression converted into CT values using the Delta-Delta Ct method. In this test, real-time PCR assays were repeated three times. Fold changes were then compared using one-way ANOVA followed by Newman-Keuls test.

**Table 1:** Relative-quantitative PCR sequences on identification of HTERT-AEC II cells.

Gene	Primers	Reaction conditions of PCR	Product sizes (bp)
HTERT	F: 5’- GTGTGCTGCAGCTCCCATTTC -3’	60°C	264
	R: 5’- GCTGCGTCTGGGCTGTCC -3’		
SP-A	F: 5’- GTGATGGGATGACTGGAGCC-3’	58°C	131
	R: 5’- TCTGAAGTCGTGGAGTGTGC-3’		
SP-B	F: 5’- ACAAGACTCTGACTGCCAGC-3’	58°C	125
	R: 5’- CACACTTTTGCCTGTCCAGC-3’		
SP-C	F: 5’- TGCCACTTTCTCCATTGGCT-3’	58°C	129
	R: 5’- AGACTTGGGATGTTCTGCGG-3’		
GAPDH	F: 5’-GATGGTGAAGGTCGGAGTGAAC-3’	60°C	103
	R: 5’-GTCATTGATGGCGACGATGT-3’		
TNF	F: 5’- CCAGGCAACTTGCTCTCTCT -3’	58°C	187
	R: 5’- GCTGAGGCACAAGCAACTTC -3’		
IL-6	F: 5’- GGCGGAGCCTTGCGTTAT -3’	58°C	118
	R: 5’-AACTGCTGTGCTTGCTTCAT-3’		

### Western blotting for cell type analysis

The expression of alveolar markers SP-A and SP-B was examined by Western blot. RIPA buffer was used for cellular protein extraction, as previously reported [23]. Then, 30 µg of cell protein from each sample was separated by 10% SDS-PAGE and transferred to a polyvinylidene fluoride membrane. After washing with Tris buffered saline with Tween 20 (TBST) and blocking with 5% skim milk in TBST at room temperature for an hour [23], membranes were separately incubated with rabbit anti-GAPDH, anti-SP-A, and anti-SP-B antibodies (Santa Cruz Biotechnology) at 4 °C overnight. Blots were incubated with horseradish peroxidase-conjugated sheep anti-rabbit IgG (Beyotime) for 2 h at room temperature and washed with TBST. Blots were then detected with enhanced chemiluminescence Western blotting reagents (Beyotime).

### Germ culture of Bacillus Calmette-Guérin and cell infection

*M. tuberculosis* of Bovis attenuated strain Bacillus Calmette-Guérin (BCG) was collected from the Lanzhou Veterinary Research Institute and cultured on Middlebrook 7H9 for 20 d. Bacterial infection tests were performed as reported by He et al. [24]. Invasion of BCG bacteria (MOI 10:1) in HTERT-AEC II cells was detected by Auramine O staining (Sigma) according to the manufacturer’s instructions. RNA extraction, RT-PCR and real-time PCR were performed as previously described. Different cells were pre-incubated in the medium for 24 h prior to mycobacterial infection. The culture medium was collected and frozen concentrated for IL-6 and TNF Western blot assays after mycobacterial invasion for 24 h, after which total cell protein was extracted as an internal control. Western blot assays were then performed as previously described. In this study, both IL-6 and TNF primary antibodies were purchased from Santa Cruz Company (IL-6, Cat: sc-7920; TNF, Cat: sc-7895).

### Statistical analysis

Data from real-time PCR analysis, cell proliferation analysis, and cell cycle analysis were analyzed by SPASS software. Fold-changes in target gene expression are presented as mean ± SEM and compared by one-way ANOVA followed by Newman-Keuls test. Values with P < 0.05 were considered statistically different.

## Results

### Primary culture of cattle type II AECs

**Figure 1:**
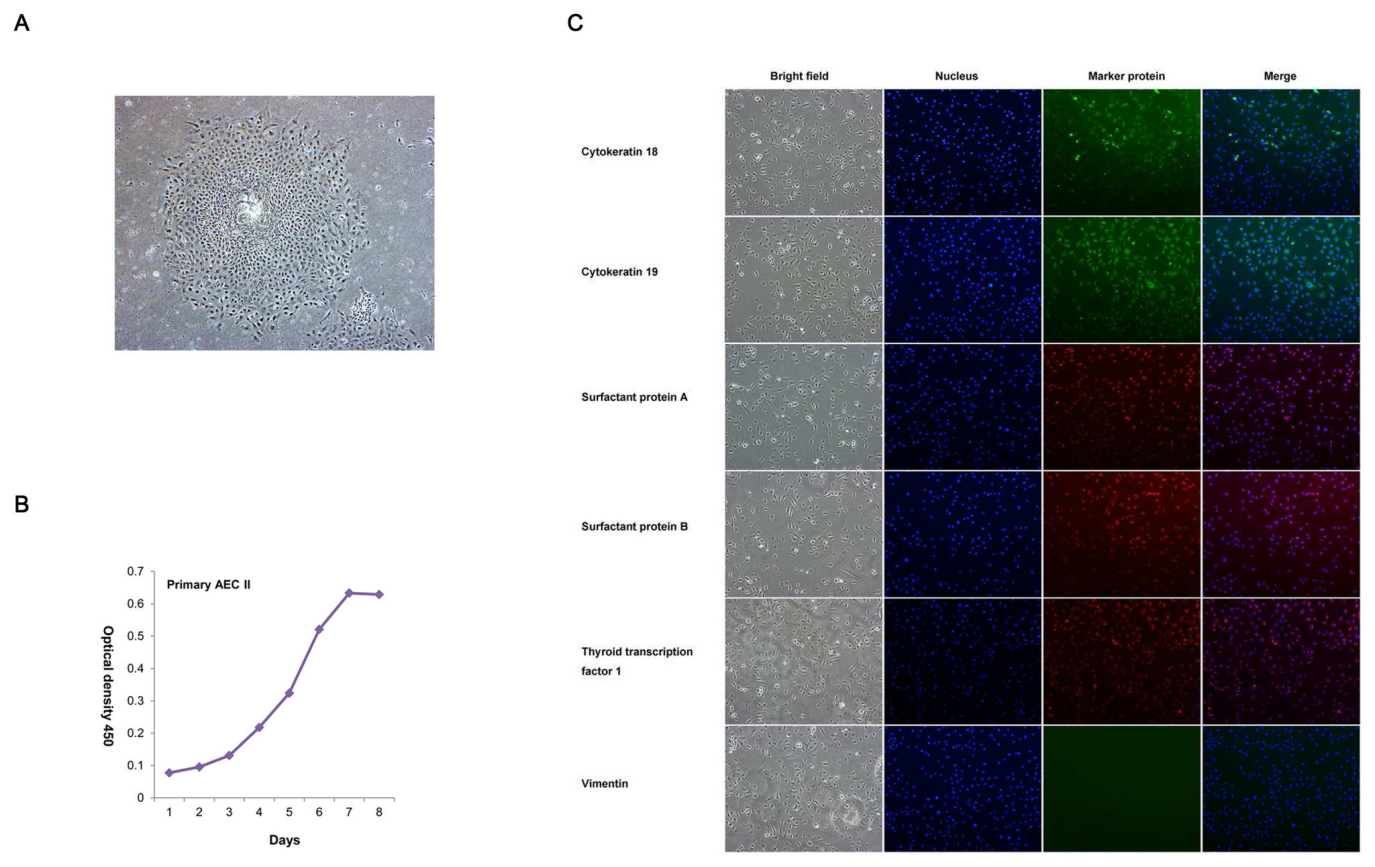
Isolation and immunofluorescence identification of AECs. A: Primary culture of AECs (200× magnification). B: Cell proliferation curve of AECs at passage 3 as determined by the WST-1 method. C: Immunofluorescence identification of AECs. The cattle AEC line isolated was determined positive by immunofluorescence testing for cytokeratin CK18, CK19, SP-A, SP-B, and TTF-1 and negative for vimentin, which confirms its AEC characteristics.

Holstein AECs were prepared by tissue culture, and AEC II cells were purified by flow cytometry. Figure 1A shows the AEC clones obtained by lung tissue culture. After purification by FACS (FITC-CD74 antibody) and passage culture, the growth curve of AEC II cells was measured by 2-(4-iodophenyl)-3-(4-nitrophenyl)-5-(2,4-disulfophenyl)-2H-tetrazolium assay at passage 3. The curve in Figure 1B shows the growth of AEC II cells.

The expression of the AEC markers SP-A, SP-B, and TTF-1 confirmed that the cells separated from the lung tissue are alveolar cells rather than fibroblasts (Figure 1C, S1). In addition, the expressions of cytokeratin 18 and 19, rather than vimentin, showed that the cultured cells have the properties of epithelial cells (Figure 1C, S1).

### Growth characteristics of HTERT-AEC II cells

**Figure 2:**
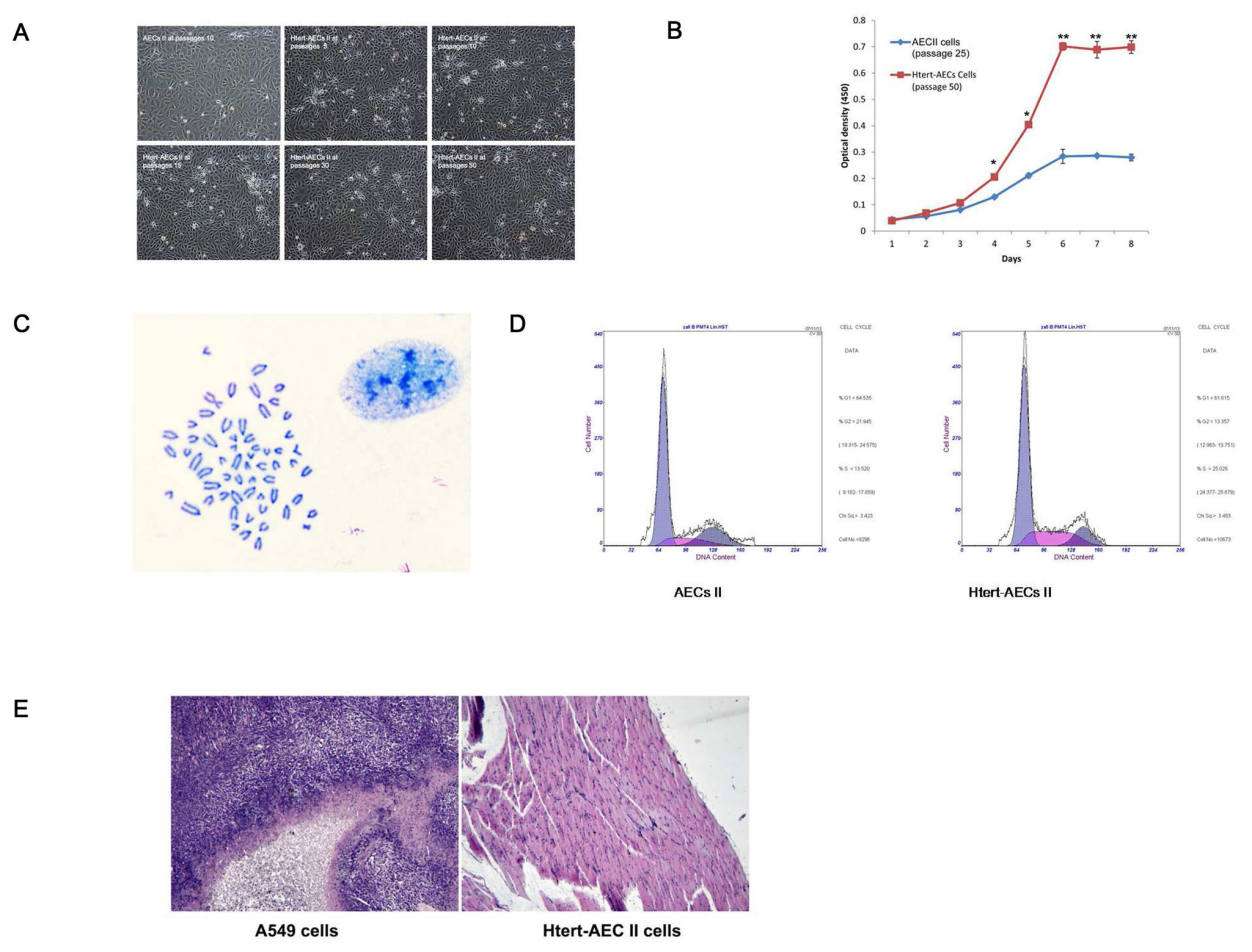
Characteristics of the HTERT-AEC II line at different passages. A: Cellular morphology of HTERT-AEC II cells at different passages. No obvious morphological differences in HTERT-AEC II cells at different passages were observed compared with normal AEC II cells. B: Cell proliferation abilities of HTERT-AEC II cells at passage 50 and normal AEC II cells at passage 25. C: Karyotype analysis of HTERT-AEC II cells at passage 50. The cell line displays the normal karyotype for cattle. D: Cell cycle comparison between primary AEC II cells at passage 5 and HTERT-AEC II cells at passage 50. E: Tumorigenicity of HTERT-AEC II cells at passage 50. Cells were injected subcutaneously into nude mice. After 2 mo, the three nude mice inoculated with A549 cells rapidly developed tumors. Histologically, tumors evolved into skeletal muscle (left, 100× magnification). However, the three nude mice inoculated with HTERT-AEC II cells did not develop tumors. Histological examination revealed normal tissue structures below the injection site (right, 100× magnification).

Immortalized HTERT-AEC II cells were evaluated by their morphological characteristics, growth characteristics, and growth cycle. We found no obvious differences in the morphological characteristics of immortalized cells at different passages compared with primary AEC II cells (Figure 2A). The proliferation potential of immortalized AEC II cells was assessed by comparison of HTERT-AEC II cells at passage 50 with AEC II cells at passage 25. The population growth rates showed significant differences. The proliferation of HTERT-AEC II cells at passage 50 clearly increased compared with that of AEC II cells at passage 25 (Figure 2B).

### Chromosomal stability of immortal HTERT-AEC II cells

To examine the karyotype and genome stability of the HTERT-AEC II cells, we analyzed the metaphase chromosomes of each AEC II line over 30 passages after HTERT transfection. HTERT-AEC II cells showed the normal cattle diploid chromosome number (2n = 60) (Figure 2C).

### Cell cycle analysis

Cells were assessed by flow cytometry to evaluate two parameters of cell proliferation. A significant difference was observed between the cell cycles of AEC II cells at passage 5 and HTERT-AEC II cells at passage 50 when they were cultured under the same conditions (Figure 2D). HTERT-AEC II cells showed an extended replicative lifespan compared with AEC II cells. The percentage of cells in S phase in HTERT-AEC II cells was higher than that in AEC II cells, which suggests that HTERT-AEC II cells have an extended replicative lifespan compared with AEC II (Table 2).

**Table 2:** Cell cycle value comparison between normal and immortalized AEC II cells.

Cell type	Cell Passage	S-phage (%)
AEC II	5	13.484±0.086
HTERT-AEC II	50	24.268±0.680*

Comparison of S-phage (%) values between AEC II and HTERT-AEC II cell lines. S-phage values were analyzed by SPASS software, presented as mean ± SEM, and compared by one-way ANOVA followed by Newman-Keuls test (*P<0.05).

### HTERT-AEC II cells did not exhibit a malignant phenotype *in vivo*

A definitive functional assay for tumorigenicity was carried out by injecting HTERT-AEC II cells at passage 50 into nude mice. Two months later, tumors were found in the positive control (A549 cells). By contrast, none of the mice that had been injected with HTERT-AEC II developed tumors, as determined by weekly inspection for over 2 mo. Samples collected from the injection site and mounted on paraffin slides confirmed this observation (Figure 2E).

### HTERT-AEC II cells maintained telomerase activity

**Figure 3:**
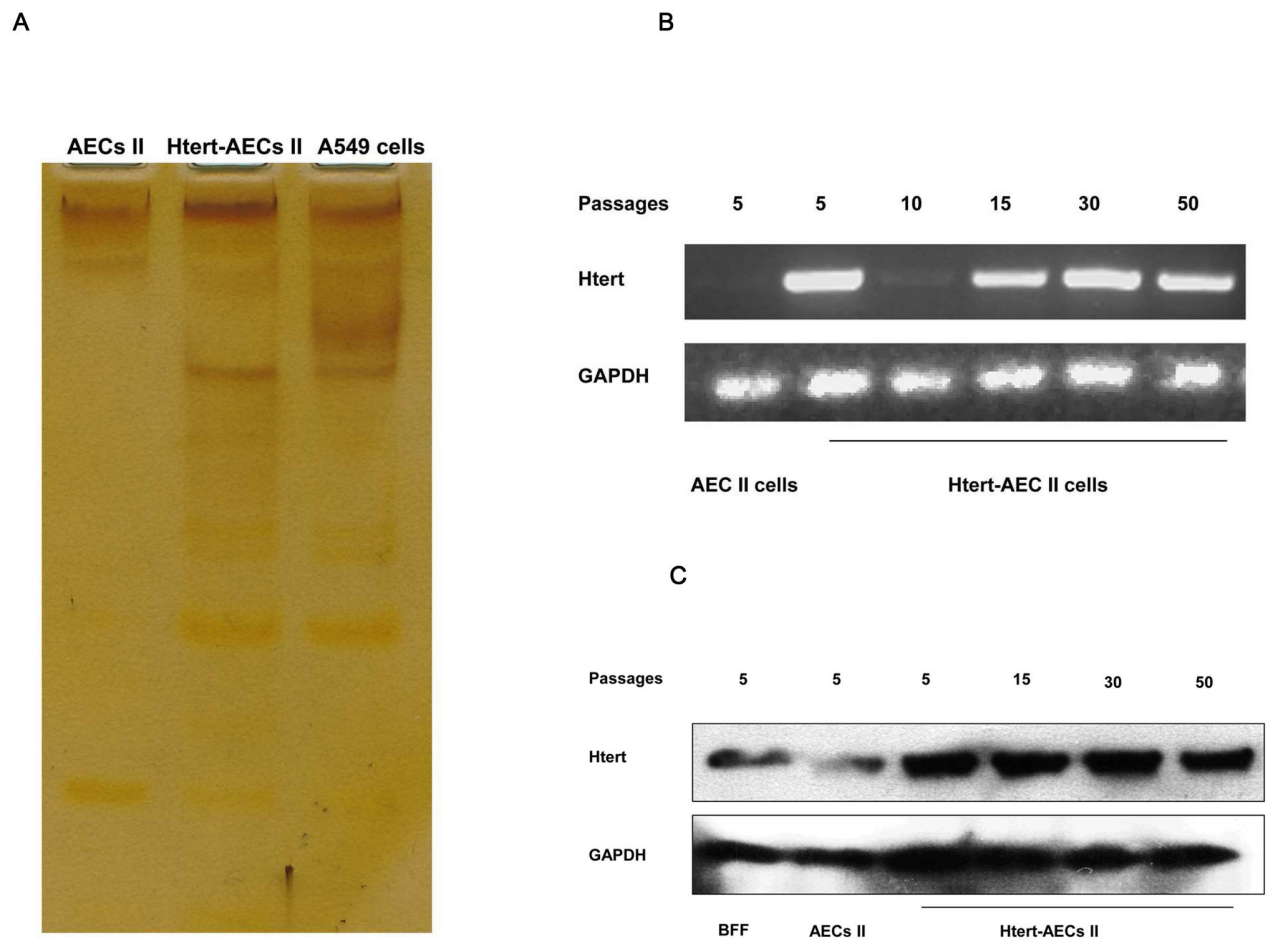
Telomerase activity detection. A: Silver staining method used to detect telomerase activity. The ladders in the gel show that HTERT-AEC II cells display telomerase activity compared with primary AEC II cells. B: RT-PCR detection of HTERT gene transcription. HTERT-AEC II cells maintained RNA levels of HTERT at passages 30 and 50. C: Western blot detection of the HTERT gene. Expression of HTERT protein is shown by HTERT-AEC II cells at different passages.

Telomerase activity was evaluated by silver staining, HTERT RT-PCR, and Western blot in HTERT-AEC II cells at different passages. HTERT-AEC II cells showed relatively higher telomerase activity than primary AEC II cells, as evidenced by silver staining results (Figure 3A). HTERT mRNA was expressed by HTERT-AEC II cells at passages 5, 10, 15, 30, and 50 but not by AEC II cells (Figure 3B). Western blot analysis further showed that the relatively high expression of HTERT in HTERT-AEC II cells compared with that in primary AEC II cells at passage 5 may the main reason that recovered and maintained telomerase activity (Figure 3C).

### HTERT-AEC II cells maintained the main biological features of AEC II cells

**Figure 4:**
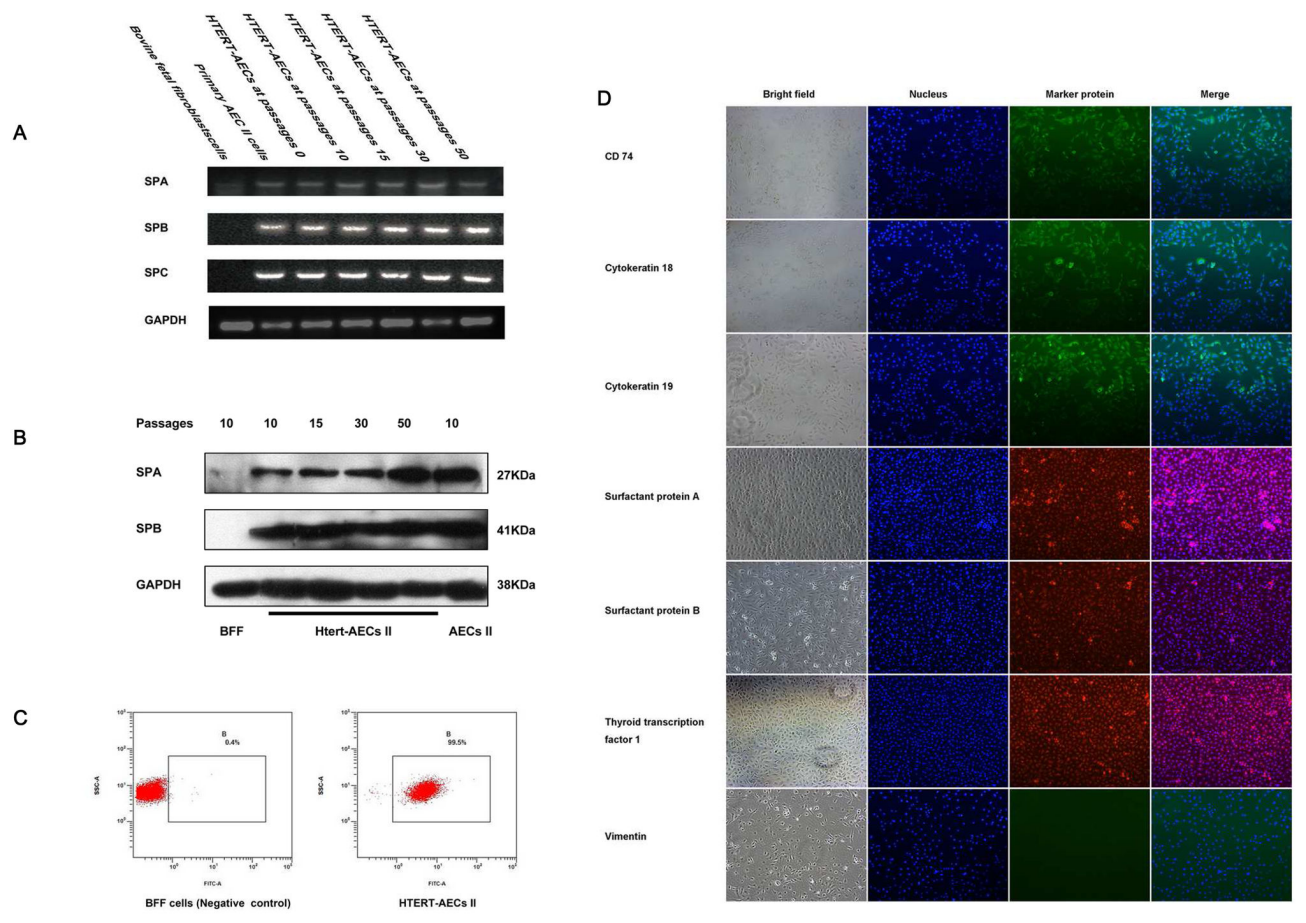
Biological features of HTERT-AEC II cells at different passages and HTERT-AEC II cells purity analysis by flow cytometry. A: RT-PCR analysis of surfactant proteins A, B, and C. RNA of surfactant proteins A, B, and C was transcribed in HTERT-AEC II cells as in primary AEC II. B: Secretion of SP-A and SP-B as determined by Western blot analysis. SP-A and SP-B proteins were expressed in immortalized AEC II. C: HTERT-AEC II cell purity was analyzed by flow cytometry using FITC-CD74 as a marker for AEC II. D: Immunofluorescence identification of HTERT-AEC II. The cattle HTERT-AEC II cell line we established was determined positive by immunofluorescence testing for CD74, CK18, CK19, SP-A, SP-B, and TTF-1 and negative for vimentin, which confirms its lung epithelial cell characteristics.

To determine whether or not HTERT-AEC II and normal AEC II cells express the same key genes, RT-PCR and Western blotting were used to detect biological features. SP-A was found in bovine fetal fibroblast (BFF), AEC II, and HTERT-AEC II cells, whereas SP-B and SP-C were only found in AEC II and HTERT-AEC II cells (Figure 4A). Western blot analysis of SP-A and SP-B showed that HTERT-AEC II cells at different passages have fundamental biological features similar to those of AEC II (Figure 4B).

### HTERT-AEC II purification analysis

HTERT-AEC II cells were stained with FITC-CD74 and then detected by flow cytometry. FACS of CD74 suggested that HTERT-AEC II cells have a high degree of purity (Figure 4C). Expression of CD74, cytokeratin 18, and cytokeratin 19, rather than vimentin, in HTERT-AEC II cells as determined by cell immunofluorescence suggested that immortalized AEC II lines retain surface markers from primary AEC II. In addition, cell immunofluorescence studies of SPA, SPB, and TTF-1 suggested that immortalized AEC II cells retain the basal physiological characteristics of primary AEC II (Figure 4D).

### Immortalized AEC II cells secreted IL-6 and TNF-α in reaction to BCG invasion

**Figure 5:**
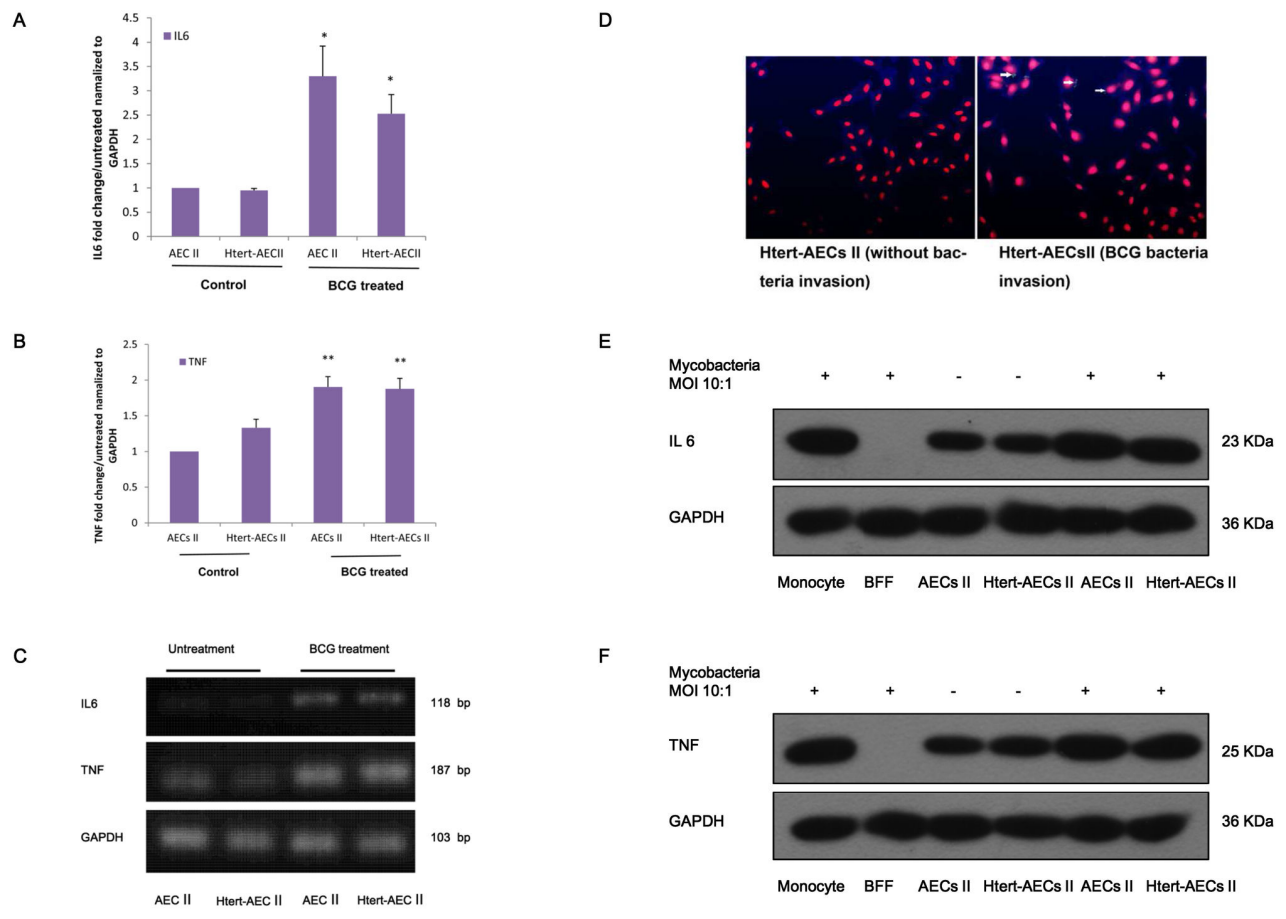
Real-time PCR analysis of TNF-α and IL-6 production by immortalized AEC II cells. A: Relative expression of IL-6 by AEC II and immortalized HTERT-AEC II cells invaded by BCG. Fold-changes in expression are presented as mean ± SEM and were compared by one-way ANOVA followed by Newman-Keuls test. *P < 0.05). B: Relative expression of TNF-α by AEC II and immortalized HTERT-AEC II cells invaded by BCG. Fold-changes in gene expression are presented as mean ± SEM and were compared by one-way ANOVA followed by Newman-Keuls test. **P < 0.01). C: Semi-quantitative expression of TNF-α and IL-6. D: BCG bacteria were detected by Auramine O staining, and the negative control included HTERT-AEC II cells that had not been infected by bacteria (left image). The yellow spot in the test result indicates the bacteria that have invaded HTERT-AEC II cells (right image). E: Western blot analysis of IL-6. IL-6 was detected from concentrated cell medium, and GAPDH was detected from cells. The positive control included RAW264.7 cells, and the negative control included BFF cells. F: Western blot analysis of TNF. TNF was detected from concentrated cell medium, and GAPDH were detected from cells. The positive control included RAW264.7 cells, and the negative control included BFF cells.

To evaluate the reaction of HTERT-AEC II cells to BCG invasion in comparison with the reaction of normal AEC II, we performed real-time PCR to detect IL-6 and TNF-α expression. Expression of TNF-α and IL-6 clearly increased during BCG invasion (Figure 5A, 5B). By contrast, no significant differences in expression were found between HTERT-AEC II and normal AEC II cells in the presence or absence of BCG invasion (Figure 5A, 5B, 5C). BCG bacteria were detected by Auramine O staining, and the negative control included HTERT-AEC II cells that had not been infected by bacteria (Figure 5D, left image). The yellow spot (arrow) shown in Figure 5D, right image, indicates the bacteria that have invaded HTERT-AEC II cells. Western blot assays showed that secretion of IL-6 and TNF increases during mycobacterial invasion (Figure 5E, 5F). No significant differences were found between AEC II and HTERT-AEC II cells.

## Discussion

The objective of this study is to establish a cattle type II AEC line, characterize its functional properties, and obtain a stable cell line for basic research on *Mycobacterium tuberculosis*. This study is significant because it contributes to the study of alveolar-specific gene functions in cattle and future research on *Mycobacterium tuberculosis*. Tissue culture methods yielded a heterogeneous population of cells with both type I and II AEC characteristics. AEC II cells express MHC class II molecules on their surface and have been proposed to be able to present antigens to CD4 T cells. By contrast, AEC I cells express podoplanin (T1a) as a marker. After expanding monoclonal cells separately, cell types and functions were identified by immunofluorescence. AEC II cells isolation procedures were based on the descriptions of Dobbs and Chen with some modifications [25,26], and the cells were further purified by FACS.

Cattle AEC II cells first appear during alveoli formation, and their differentiation is influenced by fetal lung expansion [27-30]. In the fetus, increased lung expansion promotes differentiation into the type I AEC phenotype, whereas reduced lung expansion promotes the type II AEC phenotype [31,32]. Lung expansions become progressively larger with lung development and excess AEC I may influence AEC II cell purity. Thus, fetus lung was chosen for cell culture studies in this work. Previous research suggests that the expressions of cytokeratin 18 and cytokeratin 19 are important in AEC transitions that occur during adult lung remodeling and in cultured type II cells [33]. Surfactant proteins have also been confirmed to be expressed by AEC II. As such, the expression and secretion of surfactant proteins are considered markers of mature AEC II cells [32,33]. In this study, expression of cytokeratin and surfactant proteins suggested that the AEC II cells we isolated are mature. Expression of surfactant proteins A, B, and C by AEC II cells [34] provides insights into the function of this important cell type in the lung, and the expression of cytokeratins 18 and 19 shows that the cells have epithelial characteristics. AEC II cells were isolated by flow cytometry based on extracellular staining of CD74, a specificity marker for AEC II [6,35]. In this study, expression of SP-A and SP-B in AEC II and HTERT-AEC II cells showed both types of cells have normal basal AEC functions. Expression of the extracellular marker CD74 showed that the AEC II cells were highly purified, as previously described [36]. A series of tests demonstrated that the cells we obtained were AEC II. We did not examine the AEC I cell markers T1-α and caveolin-1 [25,37] in this study because the presence of CD74 is adequate to differentiate AEC II cells from AEC I.

Previous studies have found that the morphology of AEC II cells changes as they are stimulated by alveolar damage. Tschumperlin et al. also studied the effects of deformation frequency, duration, and amplitude on AEC II viability [38-40]. In this study, the morphology of AEC II cells changed during cell proliferation or stimulation with G418 for cell selection, and then the cellular morphology displayed a typical cobblestone appearance in suitable culture medium without stimulation. Results showed that AEC II cells may have a cell repairing function characteristic that responds to injurious stimuli, similar to Clara cells of the airway [41,42].

Overexpression of HTERT in normal cells can recover telomerase and immortalize primary human and animal cells, as previously demonstrated by many researchers [18-20]. Further investigation confirms that HTERT-immortalized cells retain the properties of primary culture cells without altering their differentiated phenotype, specificity, or causing cancer-associated changes [22,43]. In the present study, HTERT-AEC II cells were established by transfection of a plasmid containing HTERT cDNA and displayed telomerase activity. However, limited telomerase activity is observed in primary AEC II cells. Immortalized AEC II cells also show no obvious cancer-associated phenotype. This study confirms that HTERT is a specialized reverse transcriptase for reconstituting telomerase activity, as previously reported [44,45].

As a barrier against mycobacterial invasion, AEC II cells not only internalize and control bacterial growth and present antigens to T cells [13,14] but also produce cytokines, chemokines, and other factors, such as IL-6 and TNF-α [15,46,47]. TNF-α and IL-6 are both involved in the Toll-like receptor signaling pathway, and they are presumably activated in a manner similar to that of other transcription factors, such as NF-kB [48]. Both factors are used to evaluate susceptibility to tuberculosis in mononuclear leucocytes [49]. Recent studies show that TNF and IL-6 are expressed in AEC II [6]. The finding that TNF-α is produced by AEC II cells indicates that AEC II and monocytes/macrophages may communicate and influence each other [6,50]. Thus, the production of TNF-α and IL-6 was chosen in this study as a criterion for the proper reaction of AEC II cells with *Mycobacteria. tuberculosis* invasion [49]. Based on real-time PCR, both AEC II and HTERT-AEC II cells are useful in future *Mycobacterium tuberculosis* research. In agreement with previous studies, this assay shows the importance of AEC II cells in *Mycobacterium tuberculosis* invasion [6,49].

## Conclusion

A stable cattle type II AEC line was established by transfecting AEC II cells with a plasmid containing the HTERT gene. The cells retained typical AEC II cell properties and had an extended replicative lifespan without neoplastic transformation. A bacterial invasion test showed that the cell line established is a potential tool for future research on the relationship between AECs and *Mycobacterium tuberculosis*.

## Supporting Information

Figure S1 **Negative control of immunofluorescence antibody.** The cattle BFF line we isolated was determined negative by immunofluorescence testing for cytokeratin CK18, CK19, SP-A, SP-B, and TTF-1 and positive for vimentin, which confirms its BFF cell characteristics.
(TIF)
