# Supplementary figures and images for: Establishment and Evaluation of a Stable Cattle Type II Alveolar Epithelial Cell Line
Source: PLoS One. 2013 Sep 26;8(9):e76036. doi: 10.1371/journal.pone.0076036 (PMC3784436; doi:10.1371/journal.pone.0076036)

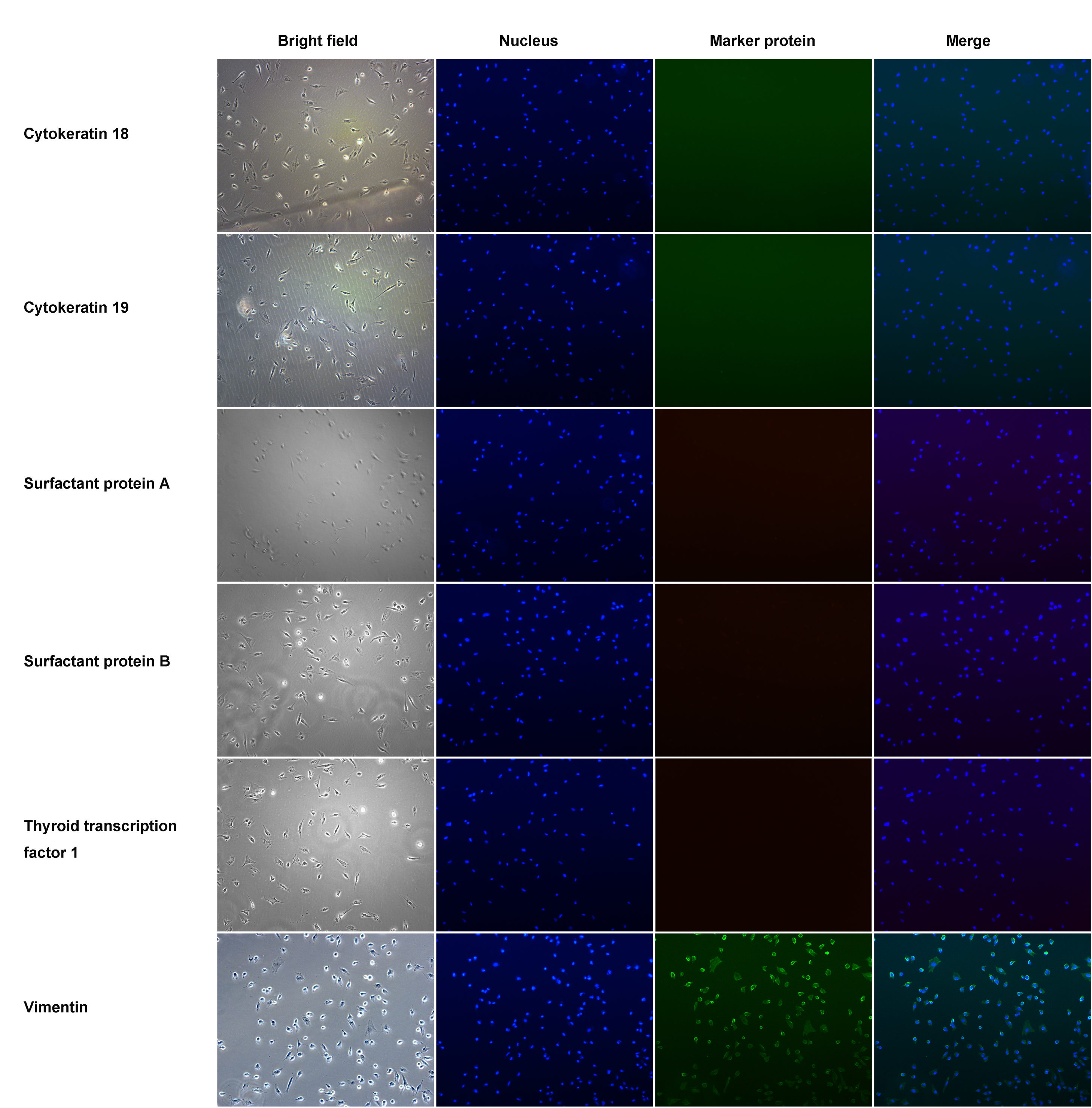

Supplement: Figure S1 — Negative control of immunofluorescence antibody. The cattle BFF line we isolated was determined negative by immunofluorescence testing for cytokeratin CK18, CK19, SP-A, SP-B, and TTF-1 and positive for vimentin, which confirms its BFF cell characteristics. (TIF) [file pone.0076036.s001.tif]
